# Supplementary figures and images for: Cross species/genera transferability of simple sequence repeat markers, genetic diversity and population structure analysis in gladiolus (Gladiolus × grandiflorus L.) genotypes
Source: PeerJ. 2023 Sep 7;11:e15820. doi: 10.7717/peerj.15820 (PMC10493085; doi:10.7717/peerj.15820)

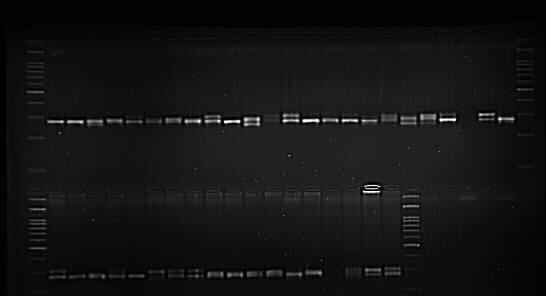

Supplement: Supplemental Information 10 [file peerj-11-15820-s010.zip › GP 13_GENOTYPE 49 - 84.jpg]

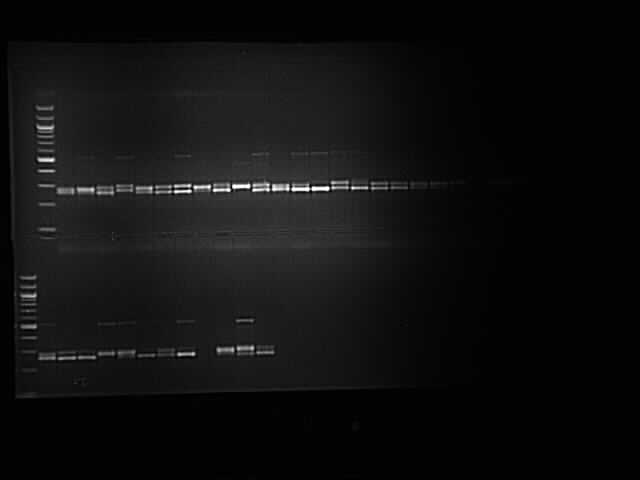

Supplement: Supplemental Information 10 [file peerj-11-15820-s010.zip › G5 Genotype 49 to 84 (1).jpg]

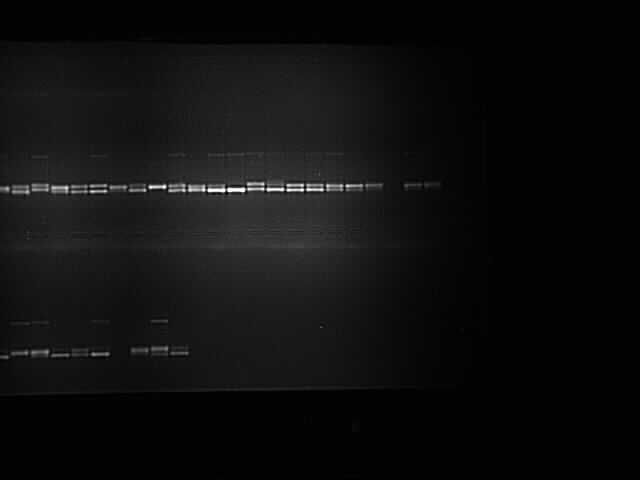

Supplement: Supplemental Information 10 [file peerj-11-15820-s010.zip › G5 Genotype 49 to 84 (2).jpg]

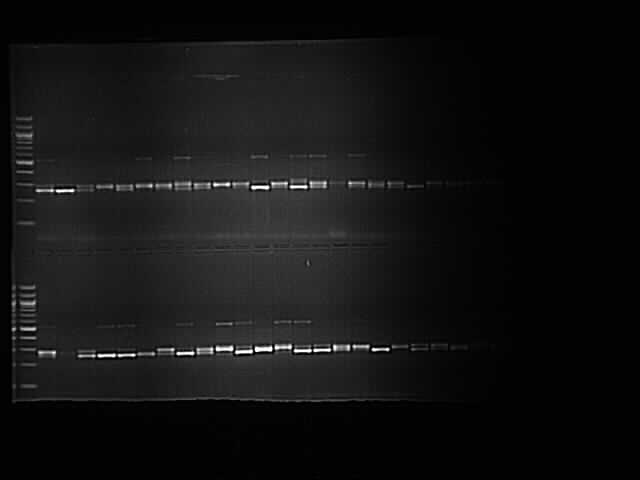

Supplement: Supplemental Information 10 [file peerj-11-15820-s010.zip › G5 genotypes 1 to 48 (1).jpg]

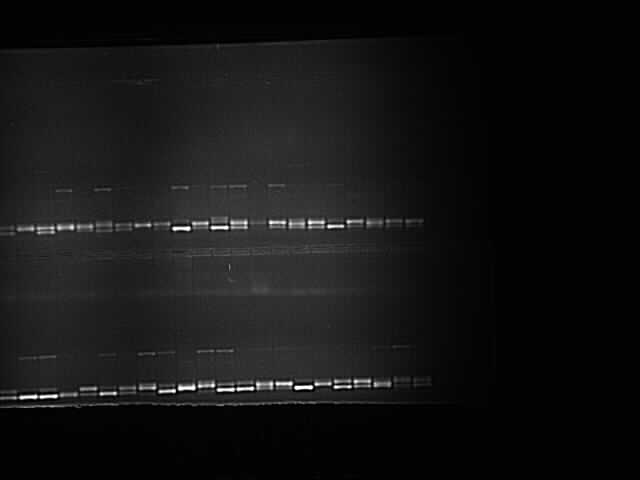

Supplement: Supplemental Information 10 [file peerj-11-15820-s010.zip › G5 genotypes 1 to 48 (2).jpg]

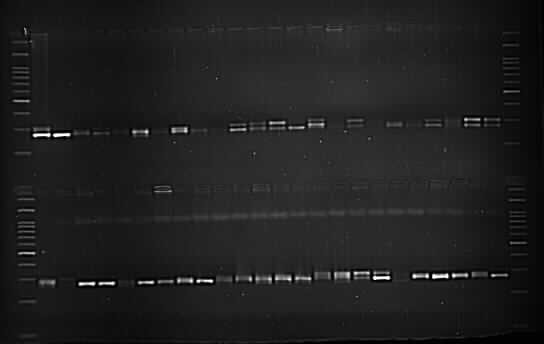

Supplement: Supplemental Information 10 [file peerj-11-15820-s010.zip › GP 13_GENOTYPE 1 - 48.jpg]
